# New Label-Free Biosensing for the Evaluation of the AX-024 Inhibitor: Case Study for the Development of New Drugs in Autoimmune Diseases

**DOI:** 10.3390/s22031218

**Published:** 2022-02-05

**Authors:** Yolanda Ramírez, María Fe Laguna, Miguel Holgado

**Affiliations:** 1 Center of Biomedical Technology, Optics, Photonics and Biophotonics Laboratory, Universidad Politécnica de Madrid, Campus Montegancedo, Pozuelo de Alarcón, 28223 Madrid, Spain; m.holgado@upm.es; 2 BioOptical Detection SL, Centro de Empresas, Campus Montegancedo, 28223 Madrid, Spain; 3 Department of Applied Physics and Materials Engineering, Escuela Técnica Superior de Ingenieros Industriales, Universidad Politécnica de Madrid, C/ José Gutierrez Abascal 2, 28006 Madrid, Spain

**Keywords:** label-free detection, point-of-care device, AX-024 inhibitor, GST-SH3.1 protein, autoimmune disease, fluorescence versus label-free detection, confocal microscopy

## Abstract

We developed a new label-free assay to evaluate the inhibition capacity of AX-024 by means of a new Point-of-Care (PoC) device for application in the development of new drugs in autoimmune diseases. The technology of PoC is based on interferometric optical detection method (IODM). For this purpose, we have optimized and developed an assay protocol whereby a Glutathione S-Transferase modified protein (GST-SH3.1), which contains a functional domain of a protein involved in T-cell activation, together with the AX-024 inhibitor has been studied. The chips used are a sensing surface based on nitrocellulose. We used streptavidin and a biotinylated peptide as links for the immobilization process on the sensing surface. The biotinylated peptide and AX-024 inhibitor compete for the same functional group of the GST-SH3.1 modified protein. When the inhibitor binds its binding site on GST-SH3.1, the biotinylated peptide cannot bind to its pocket on the protein. This competition reduces the total molecular mass of protein fixed onto the biosensor. In order to quantify the inhibition capacity of AX-024, several Ax-024:GST-SH3.1 ratios have been studied. We have compared the read-out signal for GST-SH3.1 protein not interfered by the drug, which served as a positive blank, and the response of the GST-SH3.1 modified protein blocked by the inhibitor. The technology has been correlated with confocal fluorescence microscopy.

## 1. Introduction

Autoimmune diseases are inflammatory diseases of unknown origin. They are the result of a combination of factors such as immune system disorders, environmental and genetic factors. There are millions of people affected by autoimmune diseases in the world and are associated with high morbidity and mortality [1].

Diseases such as lupus, multiple sclerosis, Crohn’s disease, autoimmune hepatitis, rheumatoid arthritis, etc., are autoimmune diseases that affect a lot of people, usually women are more affected by these disorders [2]. The immune system protects the body against invaders (viruses or bacteria). Under normal conditions, an immune response cannot be triggered against the cells of one’s own body. However, when immune cells instead of protecting, attack the other cells, an autoimmune disease occurs in the own body.

Treatment of autoimmune diseases has consisted of the use of non-selective immunosuppressive drugs, which generally offer limited clinical efficacy. Immunosuppressive drugs are used in the prevention of transplant rejection and a wide range of autoimmune diseases. The development of immunosuppressive drugs related to this type of disease is widely studied by large global pharmacies and the study of their effectiveness simulating the immune system becomes essential to evaluate their viability [3,4].

In previous works, Borroto and colleagues [5] developed a low-molecular-weight compound (AX-024) that specifically inhibits T-cell receptor TCR-triggered T cell activation. In T lymphocytes, Nck is a component of signaling pathways for T cell activation and its effector function. This protein universally coordinates cell movement, or axon guidance, connecting transmembrane receptors to multiple intracellular signaling pathways.

Nck contains three tandem Src homology 3 domains (SH3.1, SH3.2, SH3.3) and a C-terminal SH2 domain. Recent studies have shown that Nck is an important player in the function of mature T cells [6,7].

AX-024 is a compound based on blocking TCR-Nck interaction by physically binding to the Nck1-SH3.1 domain, suggesting a route to develop an inhibitor for modulating TCR activity in autoimmune and inflammatory diseases [8].

The evaluation of this type of inhibitors for autoimmune diseases is usually performed by conventional techniques such as ELISA, but the development of biosensors in recent years has made it possible to adapt this protocol to optical biosensors. Biosensors are devices capable of measuring biological response by displaying a signal depending on the concentration of the analyte in the sample to be studied. They are usually composed of a bioreceptor that is attached to a transducer and a detection system that converts the signal into an understandable signal [9,10]. There are many types of biosensors, and they can be classified in many ways, one of the most common is by the nature of the transducing signal. This signal can be magnetic, thermal, electrochemical [11], piezoelectric [12], or optical, among others.

Our research group developed a Point-of-Care device based on label-free detection [13]. The device is a compact and cost-effective Point-of-Care based on the “Increase Relative Optical Power” (IROP) principle which enhances the performance and LoD in comparison with standard high-resolution spectrometry. The transducers used are based on Fabry-Perot interferometers biophotonics sensing cells (BICELLs) [14,15,16]. In addition, in this work, an assay protocol is developed as a direct method to evaluate of inhibitor versus confocal microscopy technique to validate a new technology using a commercial and reference technique. One of the great advantages of the Point-of-Care technology presented here over the ELISA technique is that it does not require a secondary antibody for detection. Because of this, the number of test steps is reduced. In addition, the amount of sample volume required is 5 µL compared to the 50 µL needed in a well.

Furthermore, with the presented Point-of-Care technology, it is possible to measure each of the steps, which allows quality control of the assay at each of the steps, in contrast to the ELISA technique where only the final step can be measured. In addition, the multiplexing capability of the biosensors allows them to be adapted to each assay

The developed assay protocol is based on a modified protein simulating abnormal T-cells and the AX-024 inhibition efficiency over this protein is studied. The modified protein is a fusion protein between the Glutathione S-Transferase (GST) and the SH3.1 domain of NcK human gen. The GST-SH3.1 protein simulates abnormal T-cells [17,18]. In addition, in this work, an assay protocol is developed as a direct method to evaluate of inhibitor versus ELISA technique where several steps are necessary, and more reagents are used.

The objectives of this work are (i) demonstrate the capability of PoC device together with the immunoassay developed to evaluate the inhibition efficiency of new drugs, (ii) use this assay as a screening method for the study of new compounds, and (iii) validate PoC technology using commercial techniques.

## 2. Materials and Methods

### 2.1. BICELLs Fabrication and Materials

**Figure 1:**
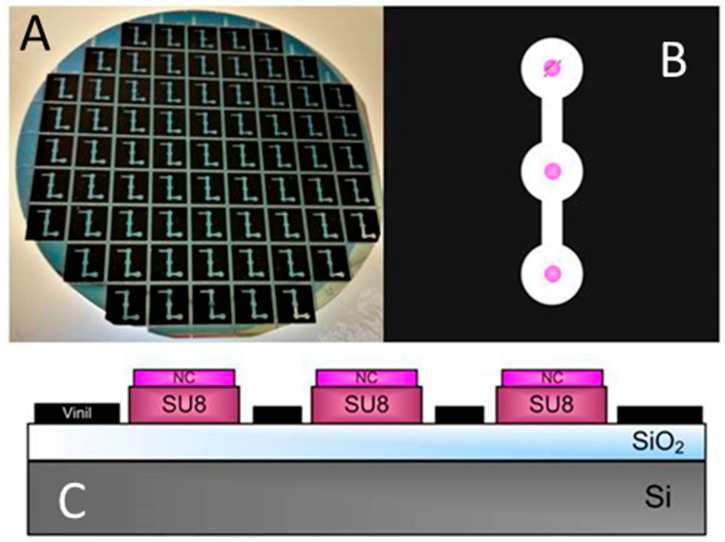
(**A**) Chip fabricated at the wafer level. (**B**) Chip made up of three sensing cells (BICELLs) and (**C**) transverse view of transducer.

The chip is fabricated by two interferometric layers based on BICELLs. These layers are composed of SiO_2_ and SU8 resist (Microchem, Westborough, MA, USA) and another thin layer of nitrocellulose (Sigma-Aldrich, St. Louis, MO, USA). It has been manufactured at the wafer level as indicated in previous works [19]. The wafer is coated by SU8 resist and after that, a thermal treatment is applied and cured by optical contact lithography with a mask. To remove the remaining resist, the wafer is developed [20]. The mask is designed to fabricate chips with three BICELLs of 100 µm in diameter. The outer layer of nitrocellulose is spun on BICELLs and exposed by DUV by means of a quartz mask which is an inverted copy of the one used in the lithography process [21]. To eliminate the remaining nitrocellulose, a developer AR-600-55 (AllResist GmbH, Strausberg, Germany) diluted in a ratio of 4:1 is used. Each chip presents three BICELLs for the study of the repeatability and reproducibility of sensing surfaces (see in Figure 1A–C).

### 2.2. Point-of-Care Device

This Point-of-Care read-out methodology is based on the measurement of IROP (%) signal, and this work was previously introduced in several publications [14,15]. This signal is defined as the quotient of the optical power of two interferometers: signal (I_Sig_) and reference (I_Ref_) interferometers in a certain optical band, given by the light source employed. Furthermore, the device has been correlated by ELISA technique using several applications [22,23].

Both interferometers are based on Fabry-Perot interferometers and are configured differently. The reference interferometer represents the minimum, and the signal interferometer shows the maximum slope within the emission wavelength range of the light emitting source. Because of this, maximum sensitivity of the measurement is achieved.

The biochemical interaction takes place in the reference interferometer which, when changing, produces a change in wavelength while the reference interferometer remains constant.

**Figure 2:**
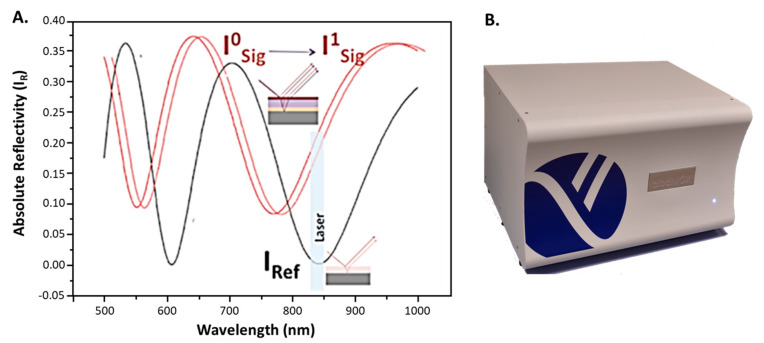
(**A**) Interference signals for reference (I_ref_) and signal (I_sign_) interferometers. (**B**) Point-of-Care device (current version).

As it is represented in Figure 2, ΔIROP (%) can be defined as the subtraction of the IROP (%) value before and after the biochemical accumulation on the sensing surface (see in Figure 2A,B) and its equation is formulated as shown in Equation (1).

(1) $$\Delta \mathrm{IROP}\;={\;\mathrm{IROP}}_{1}-{\mathrm{IROP}}_{0}=\left[\left(\frac{I_{\mathrm{Sig}}^{1}}{I_{\mathrm{Ref}}}\right)-1\right]\times 100-\left[\left(\frac{I_{\mathrm{Sig}}^{0}}{I_{\mathrm{Ref}}}\right)-1\right]\times 100\;$$

Therefore, the point of care device is based on an optical and electromechanical system. The first consists of a laser and a photodiode that performs the vertical interrogation of both interferometers with an angle of incidence of 10 degrees. The laser spot at the focal distance is about 60 µm in diameter, which allows the measurement of detection areas with a diameter less than 100 µm. The electromechanical system allows sequential measurement of the sensing surfaces in a single kit.

#### Label-Free Assay Protocol

The proposed experiment is the evaluation of AX-024 inhibition in a single step. The sensing surface of nitrocellulose allows us to biofunctionalize the chip optimally to evaluate the inhibition of compounds like AX-024. Firstly, the sensing surface will be optimized for the study of the competition between AX-024 and GST-SH3 on one specific surface.

Previously, the sensing surface is washed with Milli-Q water to remove the dust and particles from the fabrication. A biofilm of streptavidin STV (Sigma Aldrich) is bound to the sensing surface of three BICELLs [24,25]. The immobilization of streptavidin takes place by incubation of 50 µg mL^−1^ in an aqueous buffer with a volume of 5 µL for sensor surface. The incubation time is 1 h, and the temperature is 37 °C. After that, one washing step with 60 mL of distilled water is done and the chip is dried with clean and dry air. This dry air is obtained from a system consisting of an air compressor with a gun to which different particle and moisture filters have been added to ensure the quality of the air.

Then, a biotinylated peptide that recognizes the functional group of GST-SH3.1 (from Artax Biopharma, Cambridge, MA, USA) is immobilized on the sensing surface with the streptavidin biofilm. The incubation concentration is 20 µg mL^−1^, a volume of 15 µL for 30 min. The washing step is with 40 mL of distilled water.

The streptavidin-biotin interaction [26] is the strongest non-covalent biological interaction. This interaction is highly specific, rapid on-rate, and resistant to changes in temperature or pH. The Kd of streptavidin-biotin conjugate is 10^−14^–10^−15^ M. Streptavidin has four binding sites for each biotinylated peptide. This complex is highly stable and the peptide itself acts as a blocking agent on the sensing surface

**Figure 3:**
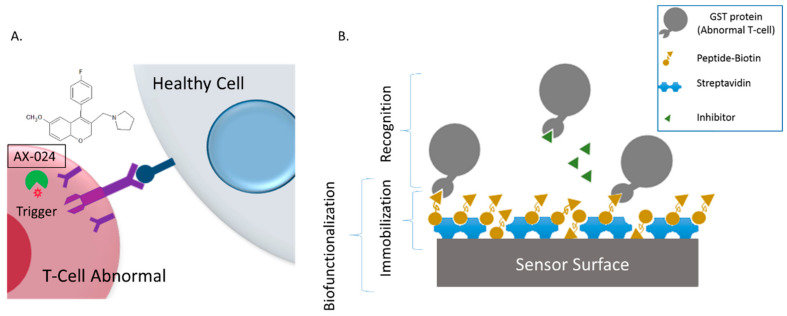
(**A**) Description of AX-024 function and (**B**) assay protocol to evaluate the AX-024 inhibition.

The sensing surface obtained has a functional group of the peptide that recognizes one specific functional group of GST-SH3.1. When a concentration of GST-SH3.1 (20 µg mL^−1^ in PBS buffer) is incubated and recognized on this surface, a recognition signal is shown (positive control). Moreover, the AX-024 inhibitor recognizes the same functional group that peptide and when there is a mixing of GST-SH3.1 and AX-024, the AX-024 compound inhibits the group of GST protein, therefore this protein is not immobilized on the sensing surface. To evaluate the AX-024 inhibitor, several concentrations of mixing of GST-SH3.1 and AX-024 are incubated on the sensing surface and the response will be lower than the positive control. This signal will be different according to the concentration of each compound in the mixing. Several molecular ratios AX-024: GST-SH3.1 are studied: 1:2, 1:1, 2.1, 4:1, 6:1, 8:1 and 10:1. Incubation conditions are 15 µL of volume, 2 h and finally, a washing step with 20 mL of distilled water (Figure 3).

### 2.3. Confocal Microscopy

The proposed experiment intends to compare the evaluation of inhibition capacity of AX-024 by using fluorescence and an optical label-free biosensing system.

Confocal microscopy is an optical imaging technique for increasing the optical resolution and contrast of a micrograph by means of using a spatial pinhole to block out-of-focus light in image formation [27]. The sample is illuminated by the laser. This light passes across the pinhole and is reflected by a dichroic mirror. After that, a small spot is focused on the sample by a microscope objective. The measure is focused on a small area of the sample. This feature permits the elimination of the fluorescence outside the spot [28,29].

#### Confocal Microscopy Assay Protocol

The assay is performed in 3 steps: immobilization, recognition and incubation with labeled antibodies.

First, the sensor surface is washed with Milli-Q water to remove any remaining particles and dust. Immobilization of streptavidin (Merck KGaA, Darmstadt, Germany) is performed by incubating a drop of 5 µL volume per cell with a concentration of 50 µg mL^−1^ in an aqueous buffer. The incubation time is 1 h in a humid environment at 37 °C. Then, the sensor is washed with 60 mL of distilled water and dried with clean and dry air.

Later, a biotinylated peptide is immobilized on the sensor surface. This peptide competes for the same functional group of the GST-SH3.1 with the AX-024. A drop of 5 µL per cell is incubated with a concentration of 20 µg mL^−1^ in an aqueous buffer for 30 min in a humid environment at 37 °C. The washing step consisted of 40 mL of distilled water and dried with clean and dry air. No blocking step is necessary since the peptide acts as a blocking agent over the sensing surface. For the recognition process, we obtained a detection surface with the peptide functional group capable of recognizing a specific functional group of GST-SH3.1. To determinate the dose-response curve of the inhibition capacity of AX-024, it is incubated 20 µg mL^−1^ of GST-SH3.1 in a PBS buffer without inhibitor with a volume of 5 µL per cell for 2 h that served as a positive blank. When AX-024 is mixed with the GST-SH3.1, the inhibitor blocks the binding site of the modified protein, avoiding the recognition by the biotinylated peptide. This fact decreases the population of GST-SH3.1 fixed onto the biosensor and hence the read-out signal. To obtain this curve, several concentrations of mixing of GST-SH3.1 and AX-024 are incubated on the kit. The ratios of ‘AX-024:GST-SH3.1’ studied are: 2:1, 1:1, 2:1 and 4:1. These ratios correspond to the following concentrations 0.29 µM, 0.58 µM, 1.16 µM and 2.32 µM. The incubation conditions are 5 µL of volume per cell for 2 h to keep it from the light and, finally, a washing step of 20 mL of distilled water is performed.

**Figure 4:**
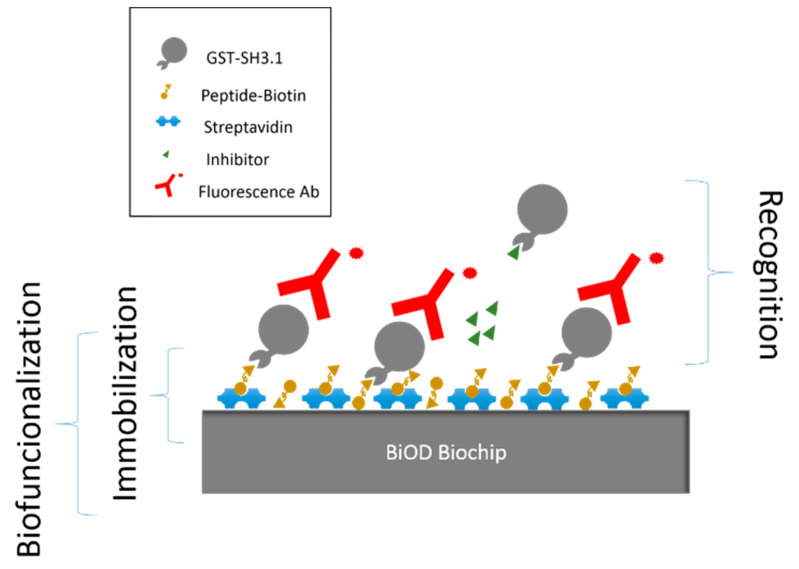
Assay protocol for fluorescence evaluation of AX-024 inhibitor.

At this point, an antibody labeled with rhodamine (Rockland) is incubated to compare both methods (fluorescence microscopy and proposed method) with a concentration of 50 µg mL^−1^ of antibody for 1 h at 37 °C in a humid environment keeping it away from the light. This model assay is shown in Figure 4.

## 3. Results and Discussion

### 3.1. Optimization of the Sensing Surface

**Figure 5:**
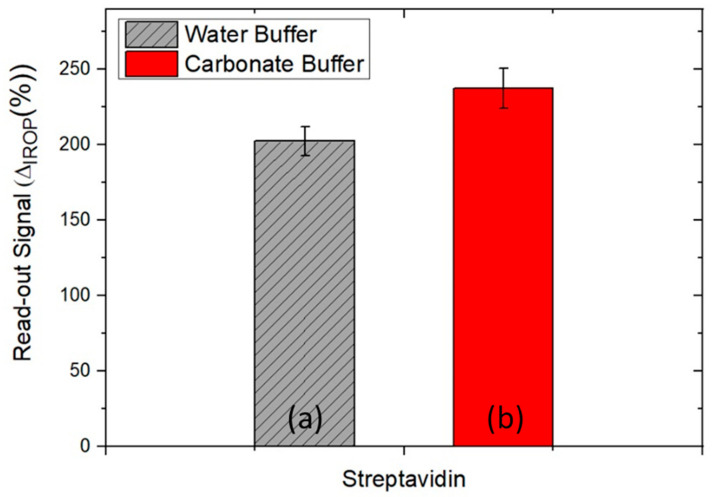
Evaluation of buffer on sensing surface of chip (a) Streptavidin in water buffer and (b) streptavidin in carbonate buffer. Error bars were calculated as the standard error of the mean (SEM) calculated as σ/√n with *n* = 9.

To optimize the sensing surface for the assay the immobilization of streptavidin with sodium carbonate buffer (pH = 9) and water buffer is carried out. A volume of 15 µL of streptavidin with a concentration of 20 µg mL^−1^ prepared with sodium carbonate buffer and deionized water buffer for one hour is incubated on the sensing surface. Afterward, the surface is washed with 60 mL distilled water and dried with pure air. In Figure 5 the signal obtained in the readout platform can be seen. The signal obtained is slightly higher for streptavidin with a carbonate buffer than streptavidin with a water buffer which could be not the only consequence of the pH but also because of the salt’s composition of the carbonate buffer that is immobilized too.

Four chips (each chip has three BICELLs) have been used for this study and the statistical standard deviation is shown.

**Figure 6:**
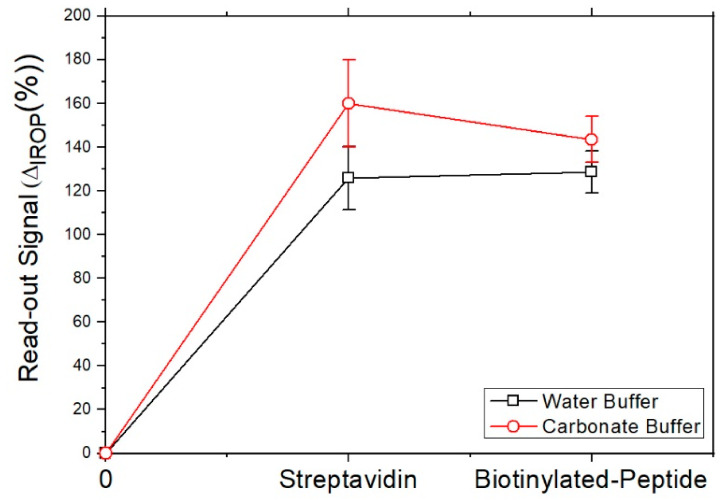
Signal obtained on sensing surface with streptavidin and biotinylated peptide. Error bars were calculated as SEM with *n* = 11.

In the second step, the biotinylated peptide is immobilized on each sensing surface with the streptavidin biofilm. Figure 6 shows the signal obtained with the immobilization of the peptide on streptavidin with carbonate buffer and water buffer. When the washing step is made, the signal decreases when the peptide is immobilized on the streptavidin surface with carbonate buffer, however, the signal of the peptide on the streptavidin surface with water buffer is as expected. In this last case, the water buffer is more stable for immobilization of streptavidin and biotinylated peptide on the sensing surface proposed than the signal obtained for carbonate buffer.

### 3.2. Evaluation of AX-024 Inhinitor with Point-of-Care Device

**Figure 7:**
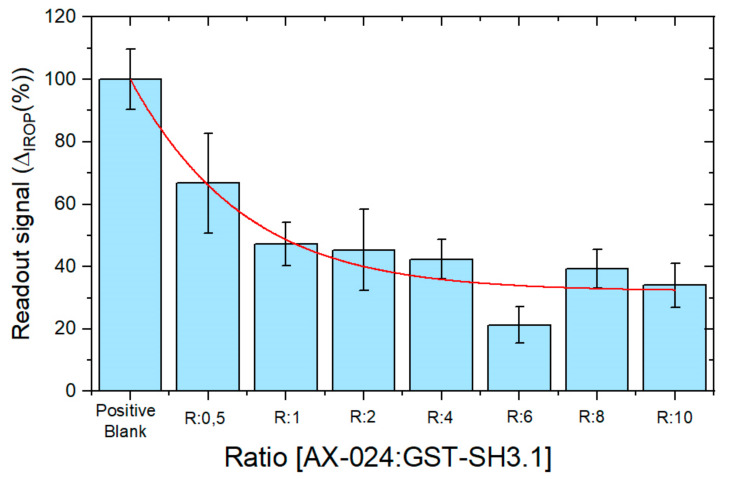
Comparison among several ratios AX-024: GST-SH3.1. The first bar from the left is the positive control.

A mixing of GST-SH3.1 and AX-024 has been incubated on the sensing surface. The positive control is the response of GST-SH3.1 without inhibitor on the sensing surface. The signal obtained for molecular ratios AX-024: GST-SH3.1: 1:2, 1:1, 2.1, 4:1, 6:1, 8:1 and 10:1 can be seen in Figure 7. The concentrations of AX-024 used in these ratios are 0.29 µM, 0.58 µM, 1.16 µM, 2.32 µM, 3.47 µM, 4.62 µM and 5.78 µM.

The first bar is the positive control with the GST-SH3.1 protein without inhibitor, this signal has been normalized to a value of 100% IROP. The following bars show the signal obtained for the different molecular ratios (AX-024: GST-SH3.1) 1:2, 1:1, 2.1, 4:1, 6:1, 8:1 and 10:1. The signal decreases with increasing AX-024: GST-SH3.1 ratio and therefore with increasing the inhibitor concentration until a certain value where there is a slow increase. The explanation for this slow increase (ratios 8:1 and 10:1) is that the compound suffers stacking from the chemical point of view and the inhibition is not effective. Error bars were calculated as SEM calculated as σ/√n in line with previous studies, where σ is the standard deviation and *n* is the sample size with *n* = 9 [30].

**Figure 8:**
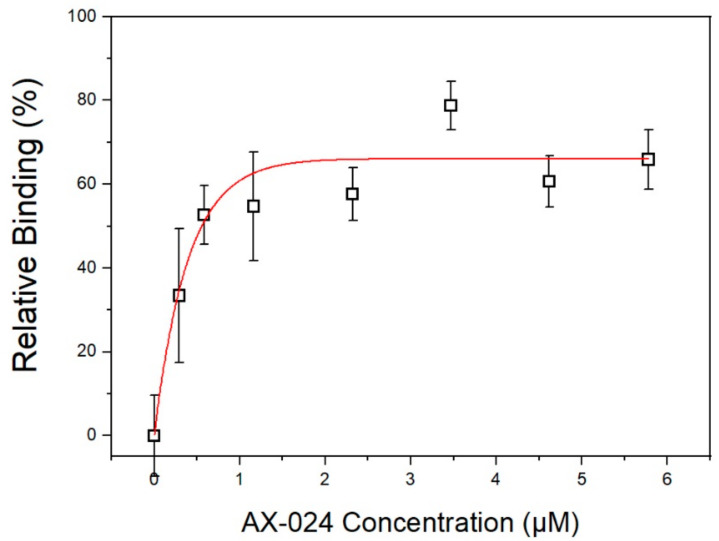
Relative binding AX-024:GST.SH3.1 versus AX-024 concentration. Error bars were calculated as SEM with *n* = 9.

According to these results, it can be represented (Figure 8) the effective relative binding between GST-SH3.1 and AX-024 obtained versus the concentration of the compound in this technique. For AX-024 concentration of 1.16 µM produces an effective binding of around 60%. Moreover, if the AX-024 concentration is tripled the effective binding increases to 80%.

### 3.3. Evaluation of AX-024 Inhibitor with Confocal Microscopy

Rhodamine labeled antibody is incubated on the kit and proceed to measure the kits in the confocal microscope.

The following bars show the signal obtained for the molecular ratios (AX-024: GST-SH3.1) 1:2, 1:1, 2.1, 4:1. The signal decrease with increasing AX-024: GST-SH3.1 ratio and therefore with increasing the inhibitor concentration until a certain value where there is a slow increase.

**Figure 9:**
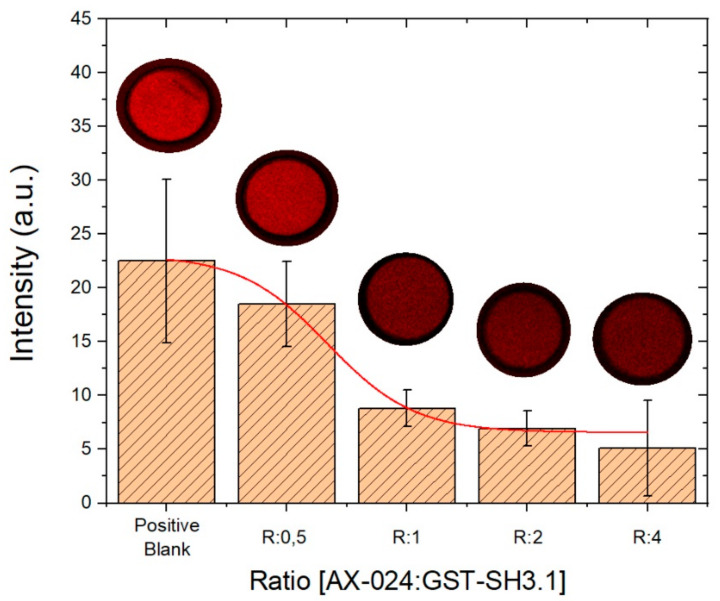
Comparison among ratios by confocal microscope. The columns show the results obtained from the intensity measurement by confocal microscopy for each of the ratios and the positive control (first column on the left). The images above the columns show the images obtained by the microscope. Error bars were calculated as the standard error of the mean SEM with *n* = 6.

As it occurs in the previous case, the first bar corresponds to the positive control. It also can be seen in Figure 9, when the concentration of AX-024 increases the intensity signal decreases. Furthermore, the error bars with the fluorescence method are bigger than with label-free proposed technology. Thus, it can be said that label-free technology is more accurate than a confocal microscope for the evaluation of AX-024 by BICELLs.

### 3.4. Comparison between Methods; Point-of-Care Device and Confocal Microscopy

**Figure 10:**
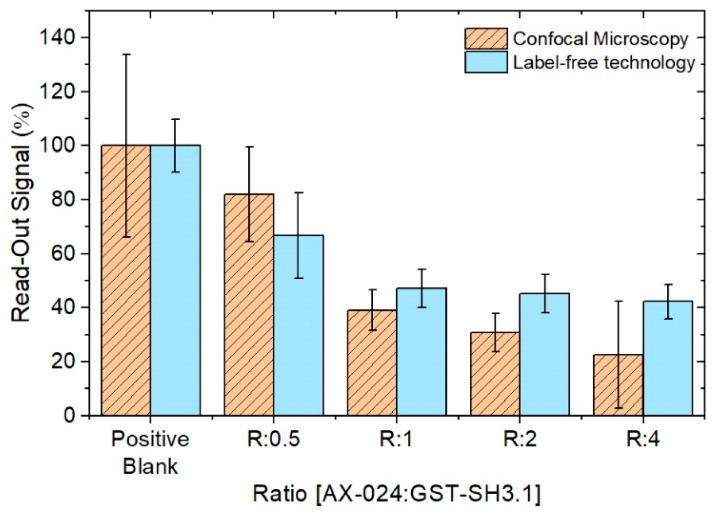
Comparison between confocal Microscope and PoC device. Values normalized to the positive blank.

To compare the results obtained by confocal microscope and Point-of-Care device, the value of the positive blank for the measures in both methods as 100% is assumed and the rest of the measures are referred to this new value. As shown in Figure 10, the results obtained for both methods are similar, the trend of the measures decreases similarly. This corroborates the proposed label-free protocol and technology.

**Figure 11:**
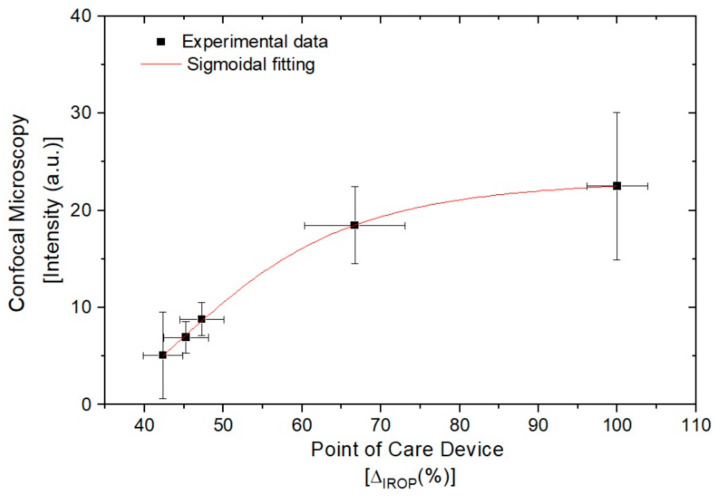
Correlation between fluorescence microscope and PoC device.

Correlation between the intensity results on confocal microscopy and the readout signal ΔIROP(%) on the Point-of-Care device is obtained. Figure 11 shows the correlation between both methods.

The measurements performed with both technologies show an excellent correlation between the results obtained with the fluorescence and the label-free based technique, demonstrating the benefits of using both technologies for the screening of immunological inhibitors in the development of new drugs. The curve fits a sigmoidal curve since saturation has been reached at the last point.

## 4. Conclusions

A new read-out methodology for one Point-of-Care device based on “Increase Relative Optical Power” IROP is developed to evaluate the AX-024 inhibition. The device is based on label-free technology, and it is a compact and cost-effective Point-of-Care compared to ELISA, its direct competitor in the market. The chips used are based on BICELLs fabricated with two interferometric layers: SiO_2_ and SU8, together with another thin layer of nitrocellulose. These chips have a very low manufacturing cost and have the capability of multiplexing so that they can be adapted to the specific needs of the assay.

An assay protocol is proposed to study the inhibition of AX-024 where the affinity of streptavidin and a biotinylated peptide is used to have an optimal sensing surface. Moreover, a modified Glutathione S-Transferase (GST-SH3.1) is used in the assay that simulates abnormal T-cells. Competition for the same functional group of the modified GST protein between the biotinylated peptide and AX-024 compound allows us to evaluate the inhibition of AX-024 drug in autoimmune diseases. The results obtained are compared with the signal of GST-SH3.1 without inhibitor. Several molecular ratios of AX-024:GST-SH3.1 are evaluated: 1:2; 1:1; 2:1; 4:1; 6.1; 8:1 and 10:1. For 1:1 molecular ratio, the inhibition of AX-024 is around 60%. If we increase the ratio of AX-024:GST-SH3.1 (6:1) the inhibition can be 80% but high concentrations of these compounds can produce stacking and the inhibition is not effective. New technology and assay protocol is presented as a screening method to evaluate new drugs for autoimmune diseases. New compounds can be evaluated and compared with each other using our technology as a screening method. The efficacy of the technique allows the evaluation of any compound with these characteristics.

In addition, the Point-of-Care device technique is compared and evaluated by using a commercial technique such as confocal microscopy. The correlation between these two techniques demonstrates the reliability of the technology to evaluate new drugs in a simpler and more robust way than with other commercial techniques. In addition, to overcome a wider range of commercially available techniques such as ELISA, the limitation of 3 measurements per kit limits the high throughput performance that must be undertaken. For this purpose, the group is already working on a new chip design in which more than 60 smaller sensing cells will be included in the future. It will allow not only to reduce the reactive and sample volume but also the costs of the assay in contrast with the economic cost that involves the ELISA.

## Data Availability

The data presented in this study are available upon request from the corresponding author. Data are not publicly available due to privacy considerations.
